# Comparison of intraoperative hemodynamic parameters between propofol- and remimazolam-based total intravenous anesthesia in patients undergoing robotic gynecologic surgery: a randomized controlled trial protocol

**DOI:** 10.3389/fphar.2026.1775804

**Published:** 2026-07-20

**Authors:** Jung Min Lee, Joohyun Lee, Se Hee Kang, Kangha Jung, Hyean Yeo, Jee Young Lee, Young Joo

**Affiliations:** 1 Department of Anesthesiology and Pain Medicine, CHA Ilsan Medical Center, CHA University, Goyang-si, Republic of Korea; 2 Department of Anesthesiology and Pain Medicine, College of Medicine, Kangwon National University, Chuncheon, Republic of Korea; 3 Department of Korean Medicine, Integrative Cancer Center, CHA Ilsan Medical Center, CHA University, Goyang-si, Republic of Korea

**Keywords:** general anesthesia, gynecologic surgery, hemodynamics, remimazolam, robot-assisted surgery

## Abstract

**Introduction:**

Propofol-based total intravenous anesthesia (TIVA) with remifentanil is widely used for general anesthesia but is frequently associated with hypotension and bradycardia. Remimazolam, an ultra–short-acting benzodiazepine, provides rapid onset and offset, with the additional advantage of reversal by flumazenil, and may result in less hemodynamic depression. Previous randomized studies have largely focused on the induction phase or the incidence of hypotension, with limited data on continuous intraoperative hemodynamic changes during laparoscopic gynecologic surgery. The study is designed to compare prospectively the intraoperative hemodynamic profiles of remimazolam-based versus propofol-based TIVA in patients undergoing robotic gynecologic surgery in the Trendelenburg position.

**Methods and Analysis:**

This prospective, single-center, randomized, single-blind, parallel-group superiority trial will be conducted at CHA Ilsan Medical Center, a secondary care university-affiliated hospital. In total, 58 adult patients (aged 19–65 years, American Society of Anesthesiologists physical status I–II) scheduled for elective robotic laparoscopic hysterectomy under general anesthesia will be enrolled and randomly assigned (1:1) to receive either remimazolam- or propofol-based total intravenous anesthesia, both in combination with remifentanil. Continuous invasive mean arterial pressure (MAP) will be monitored using a FloTrac™ sensor (Edwards Lifesciences, Irvine, CA, United States). The primary outcome will be the time-weighted average MAP (TWA-MAP), analyzed using analysis of covariance adjusted for baseline MAP. Secondary outcomes will include the area under the curve for hypertensive and hypotensive burden, as well as the incidence of adverse hemodynamic events, including hypotension, hypertension, bradycardia, and tachycardia. Generalized estimating equations (GEE) will be used to evaluate hemodynamic and respiratory parameters across predefined time points.

**Discussion:**

This trial is expected to provide prospective, randomized evidence to compare continuous intraoperative blood-pressure trajectories between remimazolam- and propofol-based TIVA during robotic gynecologic surgery. It is anticipated that the findings will clarify the relative hemodynamic stability of these anesthetic agents under the combined physiological challenges of Trendelenburg positioning and pneumoperitoneum. The findings may inform anesthetic drug selection and intraoperative blood pressure management strategies, enhancing cardiovascular stability and improving postoperative outcomes.

**Clinical Trial Registration:**

ClinicalTrials.gov, identifier NCT07251101.

## 1 Introduction

Propofol-based total intravenous anesthesia (TIVA) combined with remifentanil is widely used for general anesthesia (Lan et al., 2024). However, propofol frequently induces hypotension and bradycardia (Peng et al., 2023; Lan et al., 2024). Remimazolam, a novel ultra–short-acting benzodiazepine, has a rapid onset and offset of action, and its sedative effect can be promptly reversed with flumazenil (Peng et al., 2023; Li et al., 2025). Recent studies indicate that remimazolam may be associated with a lower incidence of hypotension than propofol, offering improved hemodynamic stability (Liu et al., 2021; Choi et al., 2023; Peng et al., 2023; Fechner et al., 2024; Li et al., 2025).

Robotic gynecologic surgery presents distinct anesthetic challenges. To facilitate the procedure, patients are positioned in the Trendelenburg position, and pneumoperitoneum is established by insufflating carbon dioxide (CO_2_) into the peritoneal cavity; these conditions produce complex hemodynamic alterations. The Trendelenburg position increases stroke volume and central venous pressure, which may subsequently elevate cardiac output and mean arterial pressure (MAP) (Falabella et al., 2007; Haas et al., 2011). Conversely, CO_2_ insufflation increases intra-abdominal pressure, leading to elevated systemic vascular resistance, reduced stroke volume, and decreased cardiac output (Darlong et al., 2012). These physiological changes may compromise cardiovascular stability, emphasizing the importance of vigilant anesthetic management.

Therefore, this randomized controlled trial will be conducted to compare the hemodynamic effects of propofol- versus remimazolam-based TIVA in patients undergoing gynecologic laparoscopic surgery in the Trendelenburg position. Propofol was selected as the comparator because it is the current standard agent for TIVA at our institution and internationally. A direct comparison will enable assessment of whether remimazolam confers clinically meaningful advantages or poses specific risks relative to established practice. The null hypothesis is that there will be no difference in intraoperative MAP between the propofol and remimazolam groups.

## 2 Methods

### 2.1 Trial design, setting, and recruitment

**FIGURE 1:**
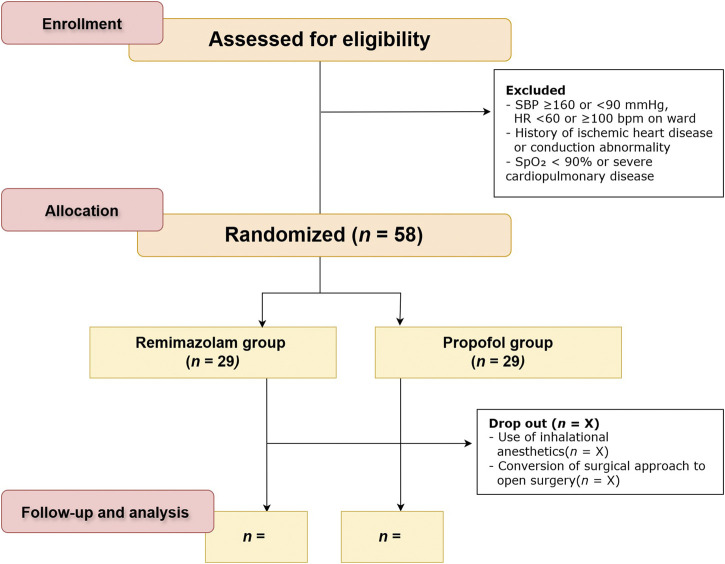
CONSORT 2025 flow diagram.

This study is a prospective, single-center, randomized, single-blind, parallel-group superiority trial that will be conducted at CHA Ilsan Medical Center. In total, 58 patients undergoing robotic laparoscopic hysterectomy will be enrolled between October 2025 and October 2026. The Consolidated Standards of Reporting Trials (CONSORT) flowchart and study timeline are presented in Figure 1 and Supplementary Table S1. This protocol was developed in accordance with the Standard Protocol Items: Recommendations for Interventional Trials (SPIRIT) guidelines, and the trial will be reported according to the CONSORT 2025 statement.

Neither patients nor the public will be involved in the design, conduct, reporting, or dissemination of the research. A plain-language summary will be provided to participants following trial completion.

No external advertising will be used for recruitment. Eligible patients will be identified at the time of hospital admission. A trained investigator will provide detailed information about the study and will obtain written informed consent before surgery.

### 2.2 Eligibility criteria

Eligible participants will be adult patients scheduled to undergo robotic laparoscopic hysterectomy under general anesthesia. Based on the inclusion criteria, patients aged 19–65 years, with a body mass index (BMI) of 18.5–35 kg/m^2^, and of American Society of Anesthesiologists Physical Status (ASA-PS) I or II will be included. The upper age limit of 65 years was selected because aging affects cardiovascular physiology and anesthetic pharmacodynamics, thereby reducing population heterogeneity (Doi et al., 2020). Similarly, the BMI range of 18.5–35 kg/m^2^ was chosen because extremes of body weight are associated with altered pharmacokinetic and pharmacodynamic characteristics that may influence hemodynamic responses to anesthetic agents (Park et al., 2018; Kim, 2021). Patients will be excluded if they present with preoperative systolic blood pressure (SBP) ≥ 160 mmHg or <90 mmHg, heart rate (HR) < 60 beats per minute or ≥100 beats per minute, hypoxemia defined as oxygen saturation (SpO_2_) < 90%, or a history of ischemic heart disease, cardiac conduction abnormalities, or severe respiratory disease, including uncontrolled asthma or chronic obstructive pulmonary disease. Additional exclusion criteria will include the administration of inhalational anesthetics during anesthesia and intraoperative conversion from robotic laparoscopic hysterectomy to open surgery.

This single-center trial will be conducted at CHA Ilsan Medical Center, a university-affiliated secondary hospital equipped with advanced robotic surgical platforms and electronic anesthesia documentation systems. All anesthesia will be administered by board-certified anesthesiologists with ≥3 years of clinical experience in total intravenous anesthesia using propofol and remimazolam infusion techniques. Participating surgeons will be experienced in robotic laparoscopic hysterectomy, each having performed >50 procedures before study initiation.

### 2.3 Study process

After receiving a comprehensive explanation of the study objectives and procedures from an attending anesthesiologist, patients who voluntarily agree to participate and provide written informed consent will undergo eligibility screening. Participants who meet the inclusion criteria will be randomly allocated to either the remimazolam group or the propofol group. All intraoperative management will be conducted independently by anesthesiologists who are not involved in outcome assessment.

### 2.4 Intraoperative monitoring and anesthetic management

Upon arrival in the operating room, all patients will undergo standard monitoring, including electrocardiography, pulse oximetry, invasive arterial blood pressure monitoring, and capnography. Preoxygenation with 100% oxygen will be performed for 3 min before induction.

For anesthesia induction, patients in the remimazolam group will receive remimazolam at 6 mg/kg/h (Lee and Kim, 2024) and remifentanil via target-controlled infusion (TCI) at an effect-site concentration (Ce) of 4 ng/mL. Patients in the propofol group will receive propofol via TCI at a Ce of 4 μg/mL together with remifentanil at 4 ng/mL. TCI will be performed using the Schneider pharmacokinetic model for propofol and the Minto pharmacokinetic model for remifentanil (Minto et al., 1997; Schnider et al., 1998). After loss of consciousness, rocuronium 0.6–0.8 mg/kg will be administered, and tracheal intubation will be performed when the train-of-four count reaches 0.

During maintenance, the remimazolam group will receive remimazolam at 1–2 mg/kg/h, whereas the propofol group will receive propofol via TCI at a Ce of 2.5–3.5 μg/mL. Anesthetic depth will be adjusted to maintain a Patient State Index (PSI) between 25 and 50 (SedLine, Masimo Corporation, Irvine, CA, United States). The Ce of remifentanil will be titrated in both groups according to intraoperative hemodynamic responses. Rocuronium will be continuously infused to maintain deep neuromuscular blockade (train-of-four count 0).

Mechanical ventilation will be set with a tidal volume of 8 mL/kg and an inspiratory-to-expiratory ratio of 1:2. The respiratory rate will be adjusted to maintain end-tidal carbon dioxide (ETCO_2_) at between 35 and 45 mmHg. A positive end-expiratory pressure of 5 cmH_2_O will be applied in all patients, and core body temperature will be maintained at between 36 °C and 37 °C.

At the end of surgery, ramosetron 0.3 mg will be administered for prophylaxis against postoperative nausea and vomiting (PONV). Neuromuscular blockade will be reversed with sugammadex 2–4 mg/kg, and flumazenil 0.3 mg will be administered in the remimazolam group to reverse residual sedative effects. Immediate postoperative analgesia will be initiated with intravenous fentanyl 50 μg, followed by standardized intravenous patient-controlled analgesia containing fentanyl, ramosetron, and nefopam.

To ensure protocol adherence, study medications will be prepared by an operating room anesthesia nurse according to a standardized protocol. Anesthesia providers will undergo pre-study training and will receive pocket reference cards outlining dosing procedures. The principal investigator will perform weekly adherence reviews, including verification of dosing accuracy and blinding, with all deviations recorded.

Standard perioperative management, including administration of intravenous fluids, vasopressors (e.g., ephedrine, phenylephrine), antibiotics, antihypertensive agents (e.g., nicardipine, labetalol), antiemetics (e.g., ramosetron), and analgesics, will be permitted according to institutional protocols. In the remimazolam group, normal saline will be used exclusively as the carrier fluid to avoid drug precipitation. The use of inhalational anesthetics, additional sedatives, including midazolam and dexmedetomidine, or investigational agents will be prohibited throughout the trial.

### 2.5 Harms and criteria for discontinuation or modification

Remimazolam and propofol are routinely used intravenous anesthetics for robotic laparoscopic surgery at our institution. Therefore, participation in our trial is not anticipated to introduce additional risk. However, hypotension and bradycardia may occur following administration of sedatives or opioids, and hypertension or tachycardia may arise in response to surgical stimuli. Hypotension (SBP <90 mmHg or MAP <65 mmHg) will be managed by reducing the remifentanil infusion rate. Severe hypotension (SBP <80 mmHg or MAP <60 mmHg) will be treated with intravenous ephedrine 5–10 mg or phenylephrine 30–100 μg. Hypertension (SBP >140 mmHg or MAP >100 mmHg) will be managed by increasing the remifentanil infusion rate, whereas severe hypertension (SBP >160 mmHg or MAP >105 mmHg) will be treated with intravenous nicardipine 300–500 μg or labetalol 5–10 mg. Bradycardia (HR < 45 beats/min) will be treated with intravenous glycopyrrolate 0.2 mg or atropine 0.25 mg, and tachycardia (HR > 100 beats/min) will be managed using intravenous esmolol 10–20 mg.

Study interventions may be modified or discontinued in cases of severe hypotension (SBP <80 mmHg or MAP <60 mmHg) unresponsive to protocol-directed vasopressor therapy, severe bradycardia (HR < 40 beats/min) unresponsive to atropine or glycopyrrolate, severe hypoxemia (SpO_2_ < 90% for more than 1 min), a severe allergic reaction to study medications, or if the attending anesthesiologist determines that continuation of the assigned intervention is unsafe. In such situations, anesthesia may be transitioned to an alternative regimen at the clinician’s discretion, and the event will be documented in the case report form (CRF).

### 2.6 Outcomes

The primary outcome of the study will be the time-weighted average (TWA)-MAP measured throughout anesthesia using an arterial line connected to a FloTrac™ sensor (Edwards Lifesciences, Irvine, CA, United States). For each participant, TWA-MAP will be calculated as the integral of MAP over elapsed time divided by the total observation duration, using the trapezoidal method. Data will be collected at approximately 1-min intervals when available, and measurements identified as technical artifacts (e.g., arterial line flushing, signal disconnection, damping artifacts, or recording errors) based on inspection of the arterial pressure waveform and corresponding clinical records will be excluded following data quality assessment. The primary analysis will compare TWA-MAP between the remimazolam and propofol groups to test the hypothesis that remimazolam-based TIVA will maintain a ≥20% higher intraoperative MAP than propofol-based TIVA.

Secondary outcomes will include the cumulative intraoperative burden of hypertension and hypotension. Hypertensive burden will be quantified as the area under the curve above the hypertension threshold (SBP ≥140 mmHg or MAP ≥100 mmHg), expressed in mmHg·min using the trapezoidal method. Hypotensive burden will be quantified as the area under the curve below the hypotension threshold (SBP <90 mmHg or MAP <65 mmHg), also expressed in mmHg·min. Group comparisons will assess whether remimazolam-based TIVA differs from propofol-based TIVA in hypertensive and hypotensive burdens. The time frame for secondary outcomes will extend from the start of induction to exit from the operating room.

In addition, intraoperative adverse hemodynamic events, including hypotension, hypertension, bradycardia, and tachycardia, will be recorded. Preoperative data will include patient characteristics, such as height, weight, age, diagnosis, scheduled surgical procedure, medical history, preoperative medication use, and baseline vital signs. Intraoperative data will include the surgical procedure performed, anesthesia duration, operative duration, degree of head-down tilt, vasopressin administration for bleeding control, estimated blood loss, fluid administration, and remifentanil dosage. Postoperative data will include post-anesthesia care unit (PACU) stay duration, PACU vital signs, and administration of additional medications, such as antiemetics and analgesics.

As a sensitivity analysis, hemodynamic and respiratory parameters will be assessed at prespecified time points to evaluate temporal changes and validate consistency with the primary outcome. Measurements will be obtained before induction (T0); every minute for 10 min from induction drug initiation (T1–T10); every 10 min for 30 min after establishment of both the Trendelenburg position and pneumoperitoneum (T20–T23); at pneumoperitoneum release (T30) and 10 min afterward (T31); and upon PACU arrival (T40), followed by every 10 min for 30 min (T41–T43). Arterial blood gas analysis will be performed at T10, T21, and T31. During anesthesia induction and maintenance, HR, SBP, diastolic blood pressure (DBP), MAP, respiratory rate (RR), SpO_2_, ETCO_2_, cardiac output (CO), cardiac index (CI), and stroke volume variation (SVV) will be recorded. In the PACU, HR, SBP, DBP, MAP, RR, and SpO_2_ will be recorded at the same prespecified intervals.

### 2.7 Sample size

A previous study reported the median and interquartile range of intraoperative MAP during total intravenous anesthesia in patients receiving remimazolam or propofol (remimazolam: 81.3 [76.9–92.5] mmHg; propofol: 76.8 [67.8–82.5] mmHg) (Tsukimoto et al., 2024). Based on these findings, we will estimate the means and standard deviations using the method described by Wan et al. (2014). The required sample size was calculated using G*Power software (version 3.1; Heinrich Heine University, Düsseldorf, Germany). With 80% power and a two-sided significance level of 0.05, 26 participants per group are required (effect size = 0.7). To account for a 10% dropout rate, we will recruit a total of 58 patients.

### 2.8 Randomization, allocation concealment, and blinding

The random allocation sequence will be generated by an independent researcher who will not be involved in patient enrollment or perioperative management. A computer-generated randomization list will be created using an online tool (Research Randomizer; https://www.randomizer.org), assigning subjects identification numbers from 1 to 58 in a 1:1 ratio (remimazolam:propofol) without replacement. Simple randomization will be applied. No stratification or fixed block sizes will be used to minimize predictability and ensure equal allocation probability.

Allocation concealment will be ensured using sequentially numbered, opaque, sealed envelopes prepared in advance by the independent anesthesiologist responsible for randomization. The envelopes will be securely stored and opened only at the time of anesthesia induction. Investigators responsible for patient enrollment and anesthesia management will not have access to the randomization sequence beforehand.

After confirming eligibility and obtaining informed consent, the independent anesthesiologist will open the next envelope immediately before induction and disclose the allocation. An operating-room anesthesia nurse will prepare the study drug according to the assigned group.

The study will be conducted in a single-blind design. Participants and surgeons will be blinded to group assignment, whereas anesthesia providers and the operating-room anesthesia nurse will not be blinded due to the practical requirements of drug preparation and infusion management. To preserve blinding, syringes containing the study drugs will be placed in syringe pumps covered with opaque panels, preventing visualization of drug appearance or pump settings. Although remimazolam and propofol differ in appearance (colorless vs. milky white) and pump dose range (1–2 vs. 2.5–3.5, respectively), these characteristics will be concealed from participants and surgeons. Both groups will receive anesthesia guided to an identical depth target (PSI 25–50) using standardized anesthetic protocols to ensure comparability aside from drug assignment.

Emergency unblinding will occur only when knowledge of group assignment is necessary for immediate clinical care. Situations that may warrant unblinding include suspected severe drug-related adverse reactions such as anaphylaxis, unexpected prolonged emergence requiring identification of the anesthetic agent, severe hemodynamic instability unresponsive to protocol-directed management, or a request from regulatory authorities or the institutional review board (IRB). In such cases, the attending anesthesiologist will initiate unblinding using the allocation record, and the time, date, reason, and personnel involved will be documented and reported to the IRB.

### 2.9 Data collection

Preoperative variables, including demographic characteristics, comorbidities, baseline vital signs, and medication history, will be collected from the electronic medical record and confirmed with the patient.

Intraoperatively, data on remimazolam or propofol infusion rate, remifentanil effect-site concentration, vital signs, continuous hemodynamic parameters (MAP, CO, CI, and SVV), and ventilator settings will be continuously recorded and synchronized with time-stamped events. Real-time physiologic data will be captured via an RS232C cable connected to the Vital Recorder software and downloaded to secure study computers for analysis (Lee and Jung, 2018).

The attending anesthesiologists will be aware of group assignment and will titrate anesthetic infusions according to hemodynamic responses and depth of anesthesia, as measured by the PSI. They will also document designated time points (T0–T43) throughout anesthesia. All intraoperative physiologic data retrieved from the Vital Recorder will be anonymized and provided to a separate research investigator blinded to group allocation. This open-label, blinded-assessment approach ensures objective and unbiased data collection and analysis.

In the PACU, vital signs and pain scores will be recorded before and after analgesic administration, along with documentation of PONV and any medications administered for symptom management.

### 2.10 Data management

All study data will be documented in standardized, paper-based CRFs developed for this trial. The CRFs will be pilot-tested in five patients before study initiation to ensure feasibility, clarity, and internal consistency. To maintain data quality, study personnel will receive pre-study training on CRF completion and data handling procedures. Duplicate checks of key variables (hemodynamic parameters and anesthetic drug doses) will be conducted, and 10% of completed CRFs will be randomly audited against source documents by an independent investigator. Any discrepancies will be resolved by consensus.

Intraoperative hemodynamic variables recorded using the Vital Recorder system will be extracted into a structured Excel file, which will serve as the source data. Relevant parameters, including MAP, HR, CO, CI, and SVV at each prespecified time point, will be transcribed into the CRF. The original Vital Recorder files will be archived as source documents linked to their corresponding CRFs.

To minimize missing data, participants will be observed continuously until discharge from the PACU. If a participant discontinues or deviates from the assigned intervention, outcome data will continue to be collected whenever feasible, including intraoperative hemodynamic variables, adverse events (hypotension, hypertension, bradycardia, and tachycardia), anesthetic drug doses, surgical characteristics, PACU duration, and postoperative vital signs. Reasons for non-adherence (e.g., conversion to inhalational anesthesia, conversion to open surgery, or discontinuation due to adverse events) and non-retention (withdrawal of consent or early termination) will be documented in the CRF. Participants who withdraw consent will be requested to permit use of data collected prior to withdrawal.

All data will be entered into a dedicated electronic database following completion of the paper CRFs. Double data entry will be conducted independently by two trained research personnel to minimize transcription errors. The database will incorporate predefined range and logic checks, and categorical variables will be coded according to a standardized data dictionary. The electronic database will be stored on a secure, password-protected institutional server with firewall protection and will be accessible only to the principal investigator and authorized study personnel. Paper CRFs and source documents will be stored in a locked cabinet within the research office. All study data will be retained for at least 3 years after publication in accordance with institutional policy and Good Clinical Practice requirements.

### 2.11 Statistical analysis

The primary outcome, TWA-MAP, will be analyzed using analysis of covariance, with group assignment as the fixed factor and baseline MAP, age, BMI, ASA-PS, and histories of hypertension and diabetes as covariates. Secondary outcomes, including hypertensive and hypotensive burden (area under the curve above or below predefined thresholds), will be compared between groups using Student’s t-test for normally distributed variables or the Mann–Whitney U test for non-normally distributed variables. Categorical outcomes, such as intraoperative hypotension, hypertension, bradycardia, tachycardia, PONV, and postoperative analgesic use, will be evaluated using the chi-square test or Fisher’s exact test, as appropriate.

Repeated-measures variables across prespecified time points (T0–T43), including HR, SBP, DBP, MAP, CO, CI, SVV, ETCO_2_, and arterial blood gas parameters, will be analyzed using generalized estimating equations (GEE) to assess group, time, and group-by-time interaction effects. An appropriate working correlation structure will be selected to account for within-subject correlations over time. As a sensitivity analysis, GEE analysis of serial blood pressure data will be performed to evaluate consistency with findings from the primary TWA-MAP analysis.

The primary efficacy analysis will follow the per-protocol (PP) principle, including all randomized participants who fully adhere to the study protocol. Participants with major protocol deviations, such as conversion to open surgery, administration of inhalational anesthetics, incomplete intraoperative hemodynamic monitoring, or early withdrawal, will be excluded from the PP analysis. Participants who discontinue the intervention due to serious adverse events will also be excluded. No intention-to-treat analysis is planned. The safety population will include all participants who receive at least one dose of the assigned anesthetic agent and will be analyzed according to the treatment actually administered.

Missing data will be minimized through prospective monitoring of CRFs. For continuous outcomes, if ≤ 5% values are missing, linear interpolation will be performed; if > 5% are missing, the participant will be excluded from the PP analysis. For repeated-measures outcomes, non-parametric generalized estimating equations will accommodate incomplete time series, allowing valid population-level inference. For categorical variables, missing values will be reported explicitly, and sensitivity analyses will be conducted to evaluate their potential influence on study findings.

### 2.12 Monitoring and interim analysis

A formal data monitoring committee (DMC) will not be established for this trial, as it is a single-center, investigator-initiated study with a relatively small sample size and short perioperative follow-up period. The safety profiles of remimazolam and propofol are well established, and both agents are routinely used in clinical practice; therefore, an independent DMC is not deemed necessary.

Interim safety oversight will be conducted by the principal investigator in collaboration with two independent senior anesthesiologists who are not involved in participant recruitment or intraoperative management. Adverse events and protocol adherence will be reviewed quarterly. Any serious adverse events will be reported immediately to the IRB and managed according to institutional policy.

No formal interim efficacy analyses are planned due to the limited sample size (n = 58) and short perioperative observation period. However, an interim review will be performed after the first 20 participants have completed the study, focusing on serious adverse events such as severe hypotension, bradycardia requiring intervention, or anaphylaxis.

No statistical stopping boundaries or alpha-spending procedures will be applied. The trial may be terminated early under the following circumstances: unexpected serious adverse events directly attributable to the study drug, safety concerns raised by the IRB, or administrative or feasibility issues such as inadequate recruitment or loss of drug supply.

Access to interim safety findings will be restricted to the monitoring team (principal investigator and independent anesthesiologists). Investigators involved in patient care, surgeons, and participants will remain blinded to group assignment during interim evaluations. The IRB will make final decisions regarding trial continuation, modification, or termination based on recommendations from the monitoring team.

### 2.13 Trial monitoring

This single-center, investigator-initiated trial involves a modest sample size and short perioperative follow-up period. Therefore, a risk-based on-site monitoring approach will be implemented rather than a formal central or remote monitoring strategy. Monitoring activities will focus on verification of participant eligibility, confirmation of informed consent documentation, assessment of the completeness and accuracy of CRFs, review of key efficacy and safety endpoints, including hemodynamic parameters and adverse events, and evaluation of protocol adherence encompassing randomization procedures, blinding integrity, and anesthetic administration. Monitoring will be conducted by the principal investigator in collaboration with an independent anesthesiologist who is not involved in direct patient care. Routine monitoring will occur quarterly, with unscheduled reviews initiated if serious adverse events or protocol deviations arise. A separate monitoring plan will not be developed due to the limited scope of this single-site study. All monitoring activities will be documented, and reports will be made available to the IRB upon request.

## 3 Discussion

Previous prospective randomized studies comparing remimazolam and propofol for general anesthesia have largely focused on the induction phase or on hypotension as the primary hemodynamic endpoint, rather than on continuous intraoperative blood pressure trends (Huang et al., 2023; Jeon et al., 2023; Xu et al., 2023; Kim et al., 2024; Kotani et al., 2024; Zhou et al., 2025). In addition, previous studies conducted in non-laparoscopic and non-gynecologic surgical settings reported that remimazolam was associated with a lower incidence of hypotension and more stable hemodynamic parameters compared to propofol.

In contrast, a recent retrospective study of patients undergoing laparoscopic surgery in the Trendelenburg position found that remimazolam infusion was associated with a higher incidence of intraoperative hypertension, whereas propofol infusion was more frequently associated with hypotension (Lee et al., 2025). These discrepant findings may reflect differences in intra-abdominal pressure, surgical positioning, and anesthetic management. However, because the study was retrospective and lacked standardized hemodynamic monitoring, the causal relationship between anesthetic selection and blood pressure fluctuations remains unclear.

Therefore, our randomized controlled trial is designed to address these limitations by prospectively comparing the hemodynamic effects of remimazolam- and propofol-based TIVA throughout the intraoperative period, including induction, maintenance, and emergence under standardized surgical and anesthetic conditions. By continuously monitoring intraoperative blood pressure and quantifying the TWA-MAP, as well as the cumulative burden of hypertension and hypotension, the study aims to provide a comprehensive assessment of hemodynamic stability during laparoscopic gynecologic surgery performed in the Trendelenburg position with pneumoperitoneum.

Propofol and remimazolam differ fundamentally in their cardiovascular pharmacodynamics. Propofol decreases sympathetic tone and induces vasodilation by modulating smooth muscle calcium flux, reducing total peripheral resistance and lowering arterial blood pressure (Marik, 2004; Soleimani et al., 2017; Sahinovic et al., 2018). Moreover, it diminishes left ventricular preload, leading to a reduction in stroke volume (Soleimani et al., 2017; Su et al., 2022). In addition, propofol suppresses the baroreceptor reflex, attenuating the compensatory tachycardic response to hypotension, which explains its propensity to cause hypotension and bradycardia during anesthesia (Marik, 2004; Soleimani et al., 2017; Su et al., 2022). In contrast, remimazolam induces hypnosis via γ-aminobutyric acid type A receptor modulation, with minimal impact on respiratory drive or hemodynamic stability (Hu et al., 2022; Ripoll et al., 2025). These mechanistic differences may account for the divergent intraoperative blood pressure patterns of the two agents, particularly under the physiologic alterations induced by Trendelenburg positioning and pneumoperitoneum.

Intraoperative blood pressure variability is influenced by multiple interacting factors. Patient-related variables such as age, baseline blood pressure, and comorbidities, including hypertension and diabetes, may affect overall hemodynamic stability. In addition, intraoperative factors, including blood loss, fluid shifts, surgical stimulation, head-down tilt during the Trendelenburg position, and pneumoperitoneum pressure, contribute to dynamic blood pressure fluctuations, as do the choice of anesthetic agents and the administration of vasoactive drugs (Kalmar et al., 2010; Hirsch et al., 2015; Cheng et al., 2025). Acknowledging these multifactorial influences, the study is designed to adjust for potential confounding effects by incorporating relevant covariates in the covariance analysis, to isolate the independent impact of the anesthetic agent on intraoperative hemodynamics. Intraoperative blood pressure instability, including hypotension and hypertension, has been associated with adverse postoperative outcomes. Hypotension is associated with myocardial injury, acute kidney injury, neurological complications such as postoperative stroke or delirium, and increased mortality, whereas hypertension may exacerbate intraoperative bleeding, impair surgical field visibility, and prolong operative time (Aronow, 2017; Wijnberge et al., 2021; Guarracino and Bertini, 2022; Szrama et al., 2023). Moreover, perioperative hypertension has been associated with an elevated risk of postoperative complications, including cardiovascular events, acute kidney injury, intracranial hemorrhage, and mortality (Aronow, 2017; Mohseni et al., 2022; Siripruekpong et al., 2022). Therefore, maintaining appropriate blood pressure control throughout surgery is essential to ensure adequate tissue perfusion while minimizing the risks of hypoperfusion and pressure-related injury.

The strengths of the study include its randomized, prospective design, standardized anesthetic and surgical protocols, and continuous arterial waveform–based hemodynamic monitoring using the FloTrac™ system. These features are expected to enhance the internal validity and reliability of intraoperative blood pressure assessment. Although a double-blind design is not feasible in this anesthetic trial, the single-blind approach—in which anesthesiologists are aware of group allocation but data collection and analysis are performed by blinded investigators—is intended to minimize bias and preserve the integrity of outcome assessment. Another strength of the study is the selection of a remimazolam induction regimen that reflects contemporary clinical practice and current safety considerations. Remimazolam induction will be performed at 6 mg/kg/h, which is within the approved dosing range and lower than the maximum recommended induction rate of 12 mg/kg/h. This dosing strategy was chosen in consideration of emerging evidence suggesting that lower induction infusion rates may reduce the risk of adverse events while maintaining satisfactory anesthetic conditions (Lee and Kim, 2024).

The study has several limitations. First, it will be conducted at a single secondary care hospital and will include only relatively healthy female patients undergoing elective gynecologic laparoscopic surgery. Therefore, the generalizability of the findings may be limited, particularly to populations with different demographic or clinical characteristics. Second, the relatively modest sample size, although determined based on an *a priori* power calculation for the primary outcome, may limit statistical power for secondary outcomes and exploratory analyses. Therefore, the findings should be interpreted with appropriate caution. Third, transient blood pressure artifacts related to surgical manipulation or device calibration cannot be entirely avoided; however, predefined data-cleaning criteria will be applied to mitigate their impact. Fourth, postoperative long-term outcomes will not be evaluated in this study. Finally, a limitation of this study is the difference in drug delivery methods between study groups. Remimazolam will be administered by continuous infusion because no externally validated PK/PD-linked target-controlled infusion model is currently available for routine clinical use, whereas propofol will be administered using a target-controlled infusion system. Although this approach reflects contemporary anesthetic practice and enhances the clinical applicability of the study design, differences in administration strategy could influence anesthetic exposure and hemodynamic responses. Future studies employing validated PK/PD-guided administration methods for remimazolam may allow more direct comparisons between anesthetic agents.

Despite these limitations, the study is expected to provide high-quality evidence regarding the differential hemodynamic effects of remimazolam and propofol during laparoscopic gynecologic surgery. The findings may help anesthesiologists optimize anesthetic drug selection and intraoperative blood pressure management strategies, enhancing hemodynamic stability and improving patient safety in minimally invasive surgery.

## Data Availability

The datasets generated and analyzed during the current study are not publicly available as data collection is planned, but are available from the corresponding author on reasonable request.
