# Supplementary material for: Comparison of intraoperative hemodynamic parameters between propofol- and remimazolam-based total intravenous anesthesia in patients undergoing robotic gynecologic surgery: a randomized controlled trial protocol
Source: Front Pharmacol. 2026 Jul 20;17:1775804. doi: 10.3389/fphar.2026.1775804 (PMC13429231; doi:10.3389/fphar.2026.1775804)
Supplement: Supplementary file 1 [file Table1.docx]

**Table S1. Participant timeline: Schedule of enrollment, interventions, and assessments.**

|  | **TRIAL PERIOD** | | | | | | | | |
| --- | --- | --- | --- | --- | --- | --- | --- | --- | --- |
|  | **Enrollment** | | **Post-randomization** | | | | | | **Post-trial** |
| **TIMEPOINT^b^** | **Day ˗ 1** | **Day 0** | **Pre-induction (T0)** | **Anesthesia induction (T1–T10)** | **Trendelenburg + CO₂ insufflation (T20–T23)** | **Deflation (T30–31)** | **End of surgery** | **PACU (T40-T43)** | **Day 1–2** |
| **ENROLLMENT:** |  |  |  |  |  |  |  |  |  |
| **Eligibility screen** | X |  |  |  |  |  |  |  |  |
| **Informed consent** | X |  |  |  |  |  |  |  |  |
| **Demographic data** | X |  |  |  |  |  |  |  |  |
| **Medical history** | X |  |  |  |  |  |  |  |  |
| **Ward vital signs** | X |  |  |  |  |  |  |  |  |
| **Randomization** |  | X |  |  |  |  |  |  |  |
| **INTERVENTION OR COMPARATOR:** |  |  |  |  |  |  |  |  |  |
| **Remimazolam group** |  |  |  | X |  |  |  |  |  |
| **Propofol group** |  |  |  | X |  |  |  |  |  |
| **ASSESSMENTS:** |  |  |  |  |  |  |  |  |  |
| **Pre-induction vital sign (MAP, HR, and SpO₂)** |  |  | X |  |  |  |  |  |  |
| **Serial hemodynamic variables (MAP, HR, SpO₂, RR, ETCO₂, CO, CI, and SVV)** |  |  |  | X | X | X | X |  |  |
| **Incidence of hypertension or hypotension** |  |  |  | X | X | X | X | X |  |
| **ABGA** |  |  |  | X | X | X |  |  |  |
| **Total anesthesia time and surgical time** |  |  |  |  |  |  | X |  |  |
| **Administered remifentanil dose** |  |  |  |  |  |  | X |  |  |
| **Fluid input or output, EBL** |  |  |  |  |  |  | X |  |  |
| **Serial hemodynamic variables (MAP, HR, SpO₂, and RR)** |  |  |  |  |  |  |  | X |  |
| **Additional medication (analgesics or antiemetics)** |  |  |  |  |  |  |  | X |  |
| **PACU LOS** |  |  |  |  |  |  |  | X |  |
| **Adverse events** |  |  |  | X | X | X | X | X |  |
| **DATA COLLECTION:** |  |  |  |  |  |  |  |  | X |

MAP, mean arterial pressure; HR, heart rate; SpO₂, oxygen saturation; RR, respiratory rate; ETCO₂, end-tidal carbon dioxide; CO, cardiac output; CI, cardiac index; SVV, stroke volume variation; ABGA, arterial blood gas analysis; EBL, estimated blood loss; PACU, post-anesthesia care unit; LOS, length of stay; CO₂, carbon dioxide.
